# Digital Twin Monitoring System for Chronic Disease Patients: A Simulation-Based Proof of Concept

**DOI:** 10.34133/csbj.0103

**Published:** 2026-06-16

**Authors:** Bushra Abbas, Saif ur Rehman Malik, Munam Ali Shah, Majid Iqbal Khan, Gautam Srivastava, Syed Atif Moqurrab, Hari Mohan Rai

**Affiliations:** ^1^ Department of Computer Science, COMSATS University Islamabad, Islamabad, 44000, Pakistan.; ^2^ Trinity College Dublin, Dublin, Ireland.; ^3^ Department of Computer Networks & Communication, King Faisal University (KFU), Al-Ahsa, Saudi Arabia.; ^4^ Sogang University, Seoul, 04107, Republic of Korea.; ^5^ Research Centre for Interneural Computing, China Medical University, Taichung, 40402, Taiwan.; ^6^ Centre for Research Impact & Outcome, Chitkara University Institute of Engineering and Technology, Chitkara University, Rajpura, 140401, Punjab, India.; ^7^ Department of Mathematics & Computer Science, Brandon University, Brandon, MB, Canada.; ^8^ School of Computing, Engineering and Creative Industries, University of Bedfordshire, Luton, UK.; ^9^ Department of Cybersecurity, Faculty of Computer Technologies and Cyber Security, International IT University, Almaty, Kazakhstan.; ^10^ Department of Computer Science, School of Engineering and Digital Sciences, Nazarbayev University, Astana, 010000, Kazakhstan.

## Abstract

The applications of digital twin (DT) technology in healthcare have shown potential to transform patient care. This paper presents a simulation-based proof-of-concept patient digital twin (PDT) for monitoring chronic diseases, namely, hypertension, diabetes, and respiratory conditions, utilizing publicly available datasets to demonstrate the potential for remote patient support. The PDT can simulate remote monitoring of a patient’s health status by integrating physiological data streams and medical records. The PDT framework was demonstrated through simulation-based experiments using publicly available datasets, enabling proof-of-concept evaluation of patient monitoring and the drug-class prediction pipeline. However, the existing PDTs for patient remote monitoring need to be shaped with sufficient information to provide a comprehensive analysis. This limitation can be addressed by incorporating the patient’s medical records along with real-time vitals in the PDT, allowing the patient’s health status to be monitored and analyzed more effectively. Through it, the patient’s health status can be monitored and analyzed effectively. We designed and simulated a PDT for chronic disease patients in a simulation-based setting to demonstrate the concept. We emulated the collection of patient vitals using public datasets to represent sensors attached to the patient’s body (physical twin) and accessed simulated medical records. By real-time simulation of the patient data, a DT of the patient (i.e., virtual replica) is created. We performed cloud-based real-time simulation so that the DT can be accessed remotely and continuously observed in a simulated environment to demonstrate the feasibility of early abnormality detection and timely intervention. The drug-class prediction module achieved 96.53% accuracy on a rule-driven synthetic dataset, demonstrating the effectiveness of the system pipeline as a preliminary validation step before proceeding to clinical evaluation.

## Highlights

• Digital twin technology has transformed healthcare by enabling remote patient care and monitoring.
• Patient digital twins (PDTs) allow healthcare professionals to provide medical assistance from a distance.
• Continuous monitoring through PDTs enables early detection of abnormalities and timely interventions.
• Integration of real-time vitals and medical records enhances the comprehensiveness of PDT analysis.
• Chronic disease patients require consistent monitoring and timely medication to manage fatal conditions.
• A PDT model for chronic disease patients was developed, combining real-time sensor data (physical twin) with digital health records.
• A cloud-based real-time simulation of patient data allows remote access to the digital twin.
• A machine-learning-based drug-class prediction system is proposed to assist in managing patient conditions remotely.
• A machine-learning-based module for drug-class prediction achieved a cross-validated accuracy of 96.53% in a simulated environment.

## Graphical Abstract

**Figure:**
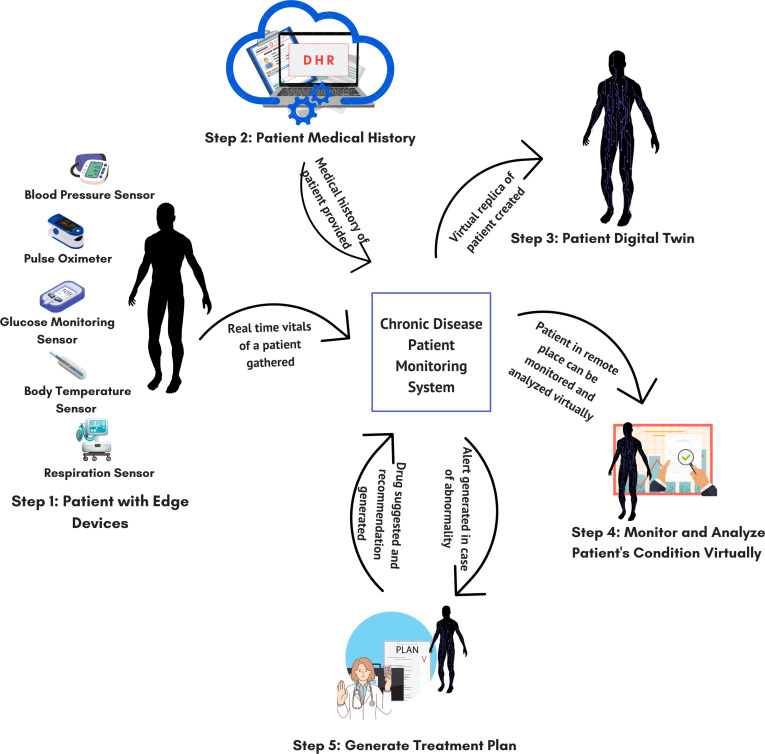

## Introduction

With advances in technology, smart devices are becoming an integral part of our lives. Healthcare technologies like smartwatches, glucose meters, fitness trackers, and smart technology clothing are being employed in daily life to achieve wellness goals. Smart healthcare services have made it easier to maintain a healthy lifestyle [1]. After the COVID-19 pandemic, the e-health sector has progressed. The demand for robust, accurate, continuous, and instantaneous remote monitoring of the patient has increased. A wireless body area network (WBAN) is a network in which sensors are attached to the human body to measure the vitals and manage the chronic disease patients’ (CDPs’) other medical conditions. However, WBANs faced several issues that need to be addressed, including wireless transmission of sensed patient data, energy efficiency, and safety management. [2–4]. The evolution of the Internet has facilitated ubiquitous connectivity in devices. The Internet of Things (IoT) refers to everything connected to the Internet. IoT-based systems are providing solutions for remote health monitoring services. Patients in remote places can be monitored by their guardians, relatives, personal medical experts, or doctors in hospitals and clinics. These systems have added ease in tracking the health status of the patient by the medical specialist remotely and responding to abnormal conditions, rather than always requiring physical presence [5]. WBAN and IoT-based monitoring systems exist in the healthcare sector to remotely assist patients. To develop such systems, a complete network needs to be designed considering latency, bandwidth, accuracy, and a lot more parameters, which has increased the complexities in such systems. This is the reason people are shifting their systems toward advanced technologies (https://www.softeq.com/blog/iot-and-digital-twins-where-the-two-technologies-meet), which help to reduce these complexities to provide accurate and instantaneous results [6,7].

The methodological contribution of this work lies in the dynamic integration of heterogeneous data sources—including real-time physiological streams and historical digital health records (DHRs)—within a unified digital twin (DT) framework. Unlike conventional remote monitoring systems that provide static alerts, our patient digital twin (PDT) architecture enables rule-based simulation of patient states within a proof-of-concept DT framework by mapping current vitals against historical baselines, allowing for personalized decision support. This approach differs from prior PDT implementations [8–11] that primarily focus on either real-time monitoring or historical data analysis, whereas our framework integrates both for predictive, patient-specific simulation.

The deployment of advanced technologies has revolutionized numerous industries such as agriculture, manufacturing, construction, and healthcare. DTs are one of them. The National Aeronautics and Space Administration has implemented the concept of DTs for the very first time by forming the DT of a spacecraft. Without launching it into space, its functionalities were tested and analyzed [12].

**Fig. 1.**
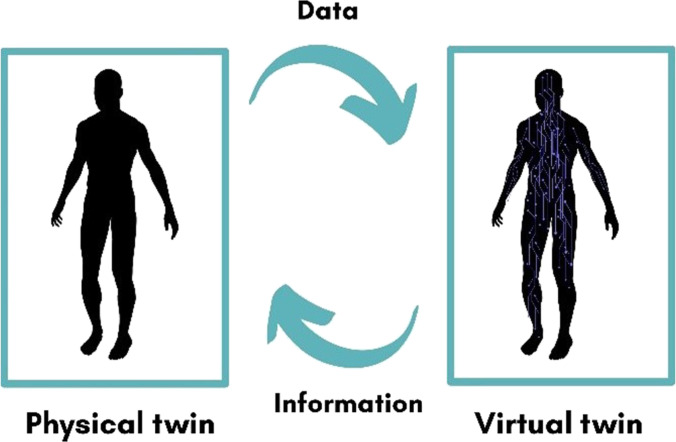
The concept of a digital twin.

**Fig. 2.**
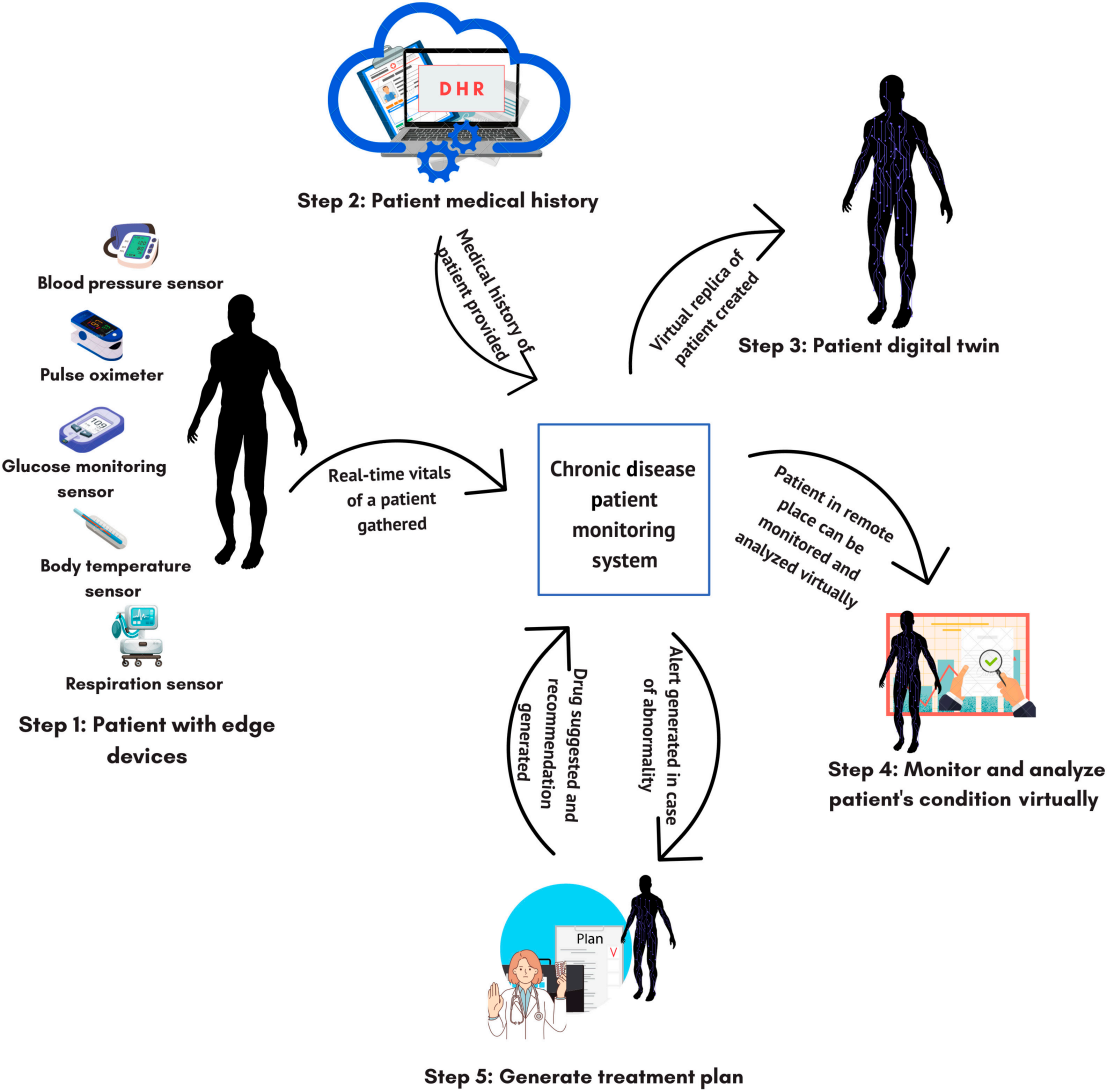
Simulation framework for the chronic disease patient monitoring system utilizing public datasets.

A DT provides instantaneous and accurate services [13,14]. It is the virtual replica of the physical entity that accurately reflects the characteristics of a physical object. It consists of 3 parts: the physical entity, the virtual entity, and the connection between them [15]. Figure 1 shows the concept of a DT. It simulates the physical object in real time and provides accurate and instantaneous results. The literature has divided it into 2 types, namely, digital twin instance and digital twin prototype. The digital twin instance is in sync with its physical twin (PT). Any variations in the PT will influence its virtual twin. The digital twin prototype creates a prototype of a physical object and performs operations on it to predict future outcomes [8]. The DT is being incorporated with other advanced technologies like artificial intelligence (AI), machine learning (ML), cyber-physical systems, and virtual reality to improve and enhance its usability. The DT can provide real-time statistics and insights to optimize the performance of PTs, perform real-time analysis, and predict future outcomes [8]. In recent years, DTs have gained popularity due to their incredible applications. Many organizations have adopted them in their respective sectors. These sectors are smart cities, manufacturing, agriculture, healthcare, automotive, energy, construction, etc. Thanks to the phenomenal abilities of DTs, especially in healthcare, they have provided several benefits. They have made it possible to create virtual replicas of entire hospitals, operating theaters, entire human bodies, a specific organ, and PDTs for monitoring vitals and patient health status. PDTs are being widely utilized for remote monitoring purposes. They reveal the health status by providing information on vitals and complete medical history instantaneously and accurately [16]. Remote monitoring of elderly patients, COVID-19 patients, and patients in the emergency room of hospitals exists [9,17,18]. It is important to monitor a CDP through a PDT to accurately manage the medical condition in a minimum time. Existing PDTs for remote monitoring are unable to provide a comprehensive analysis. Figure 2 illustrates the simulation framework for the proposed CDP monitoring system utilizing public datasets. In addition, current approaches largely remain conceptual or limited in scope, which highlights the need for proof-of-concept simulations that integrate both real-time vitals and historical medical data for chronic disease management.

Moreover, as per a report of the Centers for Disease Control and Prevention, chronic disease leads to death, and every year, millions of people die due to the severe conditions of chronic disease. In 2024, it was estimated that approximately 1.4 billion adults aged 30 to 79 years worldwide were living with hypertension, accounting for around one-third (33%) (https://www.who.int/news-room/fact-sheets/detail/hypertension) of the global population within this age group. It is important to tackle it in an appropriate way (https://www.cdc.gov/chronicdisease/index.html) to save precious lives [19]. In this regard, a recent study mentioned the potential for the application of DTs in chronic disease management [20]. Nevertheless, the majority of studies are still at an early stage, and further clinical validation is required. A DT-based monitoring system is required to monitor a CDP accurately, instantaneously, and continuously. Given these challenges, this research proposes and simulates a PDT as a feasibility study rather than a live clinical deployment. Hypertension was chosen as the initial case study due to the availability of open datasets and its high global prevalence. Importantly, while hypertension is the only condition modeled here, the proposed architecture is disease agnostic and can be extended to other chronic conditions such as diabetes and heart failure. The PDT developed in this study is evaluated in a simulated environment to explore its potential for providing comprehensive analysis and decision support while recognizing that real-world validation remains a future step.

The major contributions of this research are as follows:

• We propose and simulate a DT of CDPs to accurately and continuously monitor real-time vitals, i.e., oxygen saturation (OS), body temperature (BT), pulse rate (PR), respiratory rate (RR), blood pressure (BP), and blood glucose level (BSL), and integrate the medical history of the patient in the DT to provide a comprehensive analysis.
• Based on continuous monitoring and the current statistics, an ML-based drug-class prediction module is proposed for CDPs. It predicts appropriate drug classes by considering unstable vitals along with the respective medical background. The results obtained in this proof-of-concept simulation demonstrate promising performance for drug-class prediction (96.53% cross-validated accuracy on a synthetically constructed dataset), although clinical deployment and validation remain future work.

The rest of the paper is structured as follows: Related Work contains the literature review, which summarizes the works relevant to this research. CDP DT gives a brief overview of CDP DTs. Next, System Model and Mathematical Formulation explains our proposed work that describes the DT-based CDP formation and monitoring system. The experimentation results are discussed in Results and Discussion. Finally, the paper is concluded, and future work is suggested in Conclusion.

## Related Work

In this section, we review the existing literature on DT applications in various sectors, including healthcare, smart cities, agriculture, and manufacturing.

### Applications of DTs in healthcare

The applications of DTs in the healthcare domain are tremendous. A comparative analysis of existing PDTs is provided. Table 1 shows the comparison of the PDT of the proposed work with others. A conceptual model and a reference framework are proposed for continuous patient monitoring, data processing, and analysis according to the patient ID, gene type, and patient health records [17]. For medical resource allocation and optimization, the simulation module is utilized to adjust the beds and reception capacity of the hospital for decision-makers. The future applications of DTs are provided as an early crisis warning, an alert for medicine and its dosage, and the fall detection of an elderly patient. To handle anonymous patient emergency cases, a system model was proposed by Aluvalu *et al.* [9]. Biometric authentication is utilized to get patient data from the cloud. A patient DT is created by integrating real-time vitals, and a treatment plan is generated. The surgery simulation will minimize the operation risk for the doctor. An expert advisor interacts through the expert system to remotely plan the patient’s treatment. An empirical evaluation was done to test the proposed model. The proposed work should be validated through simulation or practical implementation. This is the main limitation of the proposed work.

**Table 1.** Comparative analysis of PDT scopes and decision support. Boldface is used to highlight the proposed work (“This research”) in comparison with prior studies in the table.

Authors/work	Real-time data	Medical history	Patient scope	Generate alerts	Integrated CDS
Aluvalu *et al*. [9]	✗	✓	Multicondition	✓	✗
Khan *et al.* [8]	✓	✗	Multicondition	✓	✗
Elayan *et al.* [10]	✓	✓	Multicondition	✓	✗
Martinez-Velazquez *et al.* [11]	✓	✓	Single (cardio twin)	✓	N/A
**This research**	✓	✓	Multicondition	✓	✓

PDT, patient digital twin; CDS, clinical decision support (including drug-class prediction and treatment suggestions); N/A, feature not within the original design scope of the specialized organ-specific twin

Several other patient-level DT applications have also been explored. Elayan *et al.* [10] proposed an intelligent context-aware framework that creates a patient DT using sensors and AI techniques to monitor health status and detect anomalies, employing long short-term memory (LSTM), multilayer perceptron, convolutional neural network, logistic regression, and support vector classifier algorithms for electrocardiogram (ECG) classification. Khan *et al.* [21] developed a real-time robot-based health monitoring system for COVID-19 patients, where the PT collects vitals via sensors to minimize direct contact. Moyaux *et al.* [22] presented an emergency department DT architecture using agent-based simulations in digital shadow, synchronized DT, and exploratory DT modes to manage patient examinations and predict future events. Thamotharan *et al.* [23] designed a human digital twin for elderly type 2 diabetes patients, integrating modules for data aggregation, diagnostics, outcome prediction, and operations management using LSTM, explainable AI, spatiotemporal attention, and learning-based model predictive control models to optimize insulin dosage. Sarp *et al.* [24] applied DTs for chronic wound care, integrating AI to predict healing progress and identify nonhealing wounds from images, with potential for further optimization.

### Applications of DTs in smart cities

DTs have applications in smart city environments to improve system-level operations, monitoring, and decision-making across urban infrastructures.

Moyaux *et al.* [22] proposed an architecture of a DT for an emergency department that relies on agent-based simulation. The proposed work operates in 1 of 3 modes: digital shadow, synchronized DT, and exploratory DT. The main purpose is to organize the patient examination process based on the severity level. The DT relies on simulation to anticipate the behavior of the PT. Monte Carlo simulations were used for predicting future possibilities in case it was not synchronized with the PT. The simulation results showed that the proposed architecture successfully tracked events, but there still exist some loopholes. This work is the very first step toward a full DT for an emergency department.

### Applications of DTs in industrial contexts

Beyond the healthcare sector, DT technology has been extensively implemented in the manufacturing industry to enhance operational efficiency and real-time decision support. Research in this domain has introduced multiphase frameworks for machine monitoring [25] and intelligent shop-floor architectures that synchronize physical and virtual spaces for production optimization [26]. Furthermore, multilayer modeling approaches have been proposed to address the complexities of system fusion [27], while the integration of big data and deep learning has been utilized to enable predictive maintenance and high-fidelity industrial simulation [27]. These industrial applications demonstrate the structural robustness of the DT concept, providing a technological foundation that can be adapted for the complex requirements of personalized patient monitoring and chronic disease management.

## CDP DT

In healthcare, the concept of DTs has been adopted to achieve efficiency and effectiveness for various purposes, including personalized medicine, monitoring, sentiment analysis, and wound management. In the case of monitoring, DTs are not designed in an appropriate way to handle the patient’s medical condition effectively. We propose a PDT that overcomes the existing limitations in remote patient monitoring and provides an effective solution to avoid emergencies, where latency and reliability are assumed ideal within the simulated environment to ensure smooth data transmission.

### Overview

In our proposed simulation-based framework, a PDT is constructed to demonstrate the feasibility of monitoring a CDP. The workflow is emulated in the following steps: First, we simulate the real-time data stream that would be generated by wearable sensors. To achieve this, physiological data representing a patient’s vitals (PR, OS, BT, BP, and BSL) are sourced from a publicly available dataset (https://www.kaggle.com/datasets/engrarri21/human-vital-signs). This dataset serves as the PT’s data input, mimicking the continuous flow of information from sensors. Second, to represent the patient’s clinical background, we create a synthetic DHR. This record is constructed using features from a separate public dataset (https://www.kaggle.com/datasets/prathamtripathi/drug-classification) containing patient demographic and historical data. This medical record is used to create a PDT to provide the doctor with complete information about the patient’s past health journey. In the third step, the PDT is created by simulating the real-time patient data obtained by wearable sensors and the medical history obtained by the DHR. The PDT will provide the complete information on the patient’s health status accurately and instantaneously. In the fourth step, the doctor monitors and analyzes the current patient’s health status remotely through the PDT. The real-time figures and simulation are displayed, which provide complete insights into the patient’s health condition. In the fifth step, an ML-based drug-class prediction module is proposed. It generates decision-support outputs by predicting appropriate drug classes and suggesting precautions in case of abnormality to help mitigate emergencies. An alert is also generated to inform the patient of an abnormal health status.

### Physiological sensor mapping for the proof-of-concept PDT

To ensure clinical relevance and meaningful simulation, the study focuses on chronic conditions that can be effectively monitored via 6 vital signs. Table 2 maps the selected sensors to their corresponding clinical parameters and target conditions, enabling precise evaluation of the PDT framework in these contexts.

**Table 2.** Mapping of sensors to chronic disease monitoring

Sensor	Vital sign	Clinical utility/target conditions
Max30102	SpO_2_, pulse rate (PR)	Respiratory conditions (COPD and asthma), heart failure
LM35	Body temperature	Infection monitoring, inflammatory conditions
Glucometer	Blood glucose level (BGL)	Diabetes mellitus (types 1 and 2)
AD8232	Blood pressure	Hypertension, cardiovascular disease
Respiration sensor	Respiratory rate (RR)	Respiratory distress, sleep apnea

COPD, chronic obstructive pulmonary disease

### Components

This section describes the components of the proposed model. To provide the technical details of the proposed model, we divide the proposed model into the following sections.

#### Physical twin

The DT of a physical, real-world entity is modeled by simulating its physiological behavior and data generation patterns. In the proposed proof-of-concept system, the PT is represented by emulated data streams sourced from publicly available datasets (https://www.kaggle.com/datasets/prathamtripathi/drug-classification) rather than a live clinical deployment. To demonstrate the system’s potential, the simulation framework models the data outputs that would be generated by various sensors—including Max30102 (SpO_2_/pulse), LM35 (temperature), and AD8232 (ECG/BP). While a physical implementation would involve attaching these sensors to the patient’s body, this simulation-based study emulates the continuous measurement and cloud-based transmission of patient vitals (PVs) (BP, PR, OS, BT, BSL, and RR) to facilitate the creation and synchronization of the cloud-based PDT.

#### Patient’s medical history

To identify the abnormal condition accurately, there is a need to provide the medical history of the patient. This is the way to appropriately suggest the medicine and control the abnormal patient condition from a distance. The patient’s medical history will be accessed from the DHR. It contains the patient’s personal information, disease information, data on all lab tests, data on medication, and diet information. It will be retrieved from the DHR and fed into the cloud-based DT.

#### Patient DT

Existing PDTs for patient monitoring are often limited by the lack of dynamic integration between real-time physiological data and longitudinal medical history. In our proposed PDT framework, simulated patient data are integrated with historical records for comprehensive analysis. This integration enables the generation of automated medication recommendations and precautionary alerts to mitigate emergency risks. The framework consists of 2 core modules: a real-time data simulation module and a DHR integration module. The data simulation module emulates the aggregation of PVs—specifically OS, BP, BT, PR, RR, and BSL—modeled after inputs from wearable sensors. Within the PDT, these vitals are monitored against evidence-based clinical thresholds. The threshold for normal OS is 95% to 100%; values below 90% indicate hypoxemia. For BP, according to authoritative clinical guidelines (https://www.who.int/news-room/fact-sheets/detail/hypertension), a reading below 90/60 mmHg indicates hypotension (low BP), while one less than 120/80 mmHg is considered the normal range. An elevated range is defined as systolic 120 to 129 mmHg with diastolic BP less than 80 mmHg. Hypertension stage 1 is defined as systolic blood pressure (SBP) between 130 and 139 mmHg or diastolic pressure between 80 and 89 mmHg. Hypertension stage 2 is defined by a systolic reading of 140 mmHg or higher and/or a diastolic reading of 90 mmHg or higher. For PR, the normal resting range is 60 to 100 beats per minute (bpm); below 60 bpm is considered bradycardia (low range), and above 100 bpm indicates tachycardia (high range). BT is monitored against a normal range of 97.8 to 99.1 °F (36.5 to 37.3 °C). Values outside this range are flagged as abnormal. The normal range for RR is 12 to 20 breaths per minute; deviations below 12 or above 20 breaths per minute indicate respiratory distress. For BSL, the normal fasting range is 70 to 100 mg/dl; below 70 mg/dl is considered hypoglycemic, 100 to 125 mg/dl indicates prediabetes, and 126 mg/dl or higher indicates diabetes. These vital signs are interpreted within the context of the patient’s health record, which is retrieved from the simulated DHR. By combining real-time vitals with the patient’s clinical history, the PDT provides a comprehensive analysis for remote decision support.

#### Monitoring and analysis

CDPs will be monitored by the personal expert remotely through DTs. The patient’s personal expert can monitor and track the patient’s medical condition. Moreover, the abnormality of vitals and symptom severity tracking are managed, and on this basis, alerts are generated. The alerts are generated for both the patient and the expert.

#### ML-based drug-class prediction module

To simulate automated decision support, an ML-based drug-class prediction module is proposed to predict suitable drug categories and precautionary actions. This module is designed to assist clinicians by identifying potential treatment pathways in a simulated remote setting. We utilized a decision tree ML technique to implement this module. The decision tree operates on a supervised learning framework, categorizing and predicting target drug classes by learning interpretable decisive rules inferred from physiological features and historical patient data. This provides a transparent and rule-based foundation for clinical decision support within the proof-of-concept simulation.

## System Model and Mathematical Formulation

This section presents the detailed modeling of the PDT framework, including formal definitions for simulated patients, vital signs, sensor mappings, and alert logic. The mathematical formulation provides a structured framework to represent how physiological data streams are captured, synchronized with historical health records, and processed to generate real-time clinical alerts and automated drug-class predictions within the proof-of-concept simulation environment. This formalization ensures a consistent and reproducible logic for evaluating the PDT’s performance across different patient profiles.

### Symbol and notation table

Table 3 summarizes the symbols and notations used in the PDT simulation model.

**Table 3.** Symbol and notation table

Symbol	Definition
*P*	Set of simulated patients $\left\{p_{1},{\;p}_{2},\ldots ,{\;p}_{N}\right\}$
*χ*_i_	Set of real-time vital signs for patient *i* (OS, BSL, PR, RR, BT, and BP)
*T_χ_*	Threshold values for vital signs
*d*	Simulated digital health record (DHR) data
*e_i_*	Abnormal vital alert (AVA) for patient *i*
*λ_i_*	Disease-specific alert (DSA) for patient *i*
*DT_i_*	Digital twin instance for patient *i*

OS, oxygen saturation; BSL, blood glucose level; PR, pulse rate; RR, respiratory rate; BT, body temperature; BP, blood pressure

### Patient-digital twin mapping

A summary of publicly available datasets used for PDT simulation is given in Table 4. Each simulated patient is associated with a DTI containing their vital signs and historical medical data:

$${\mathrm{DT}}_{i}=\left\{\chi_{i},\;o_{i}\right\},\;\forall p_{i}\in P$$ (1)

where $o_{i}$ represents the simulated patient history from the DHR dataset. Each patient has exactly one DT:

$$\left|{\mathrm{DT}}_{i}\right|=1,\;\forall p_{i}\in P$$ (2)

**Table 4.** Summary of publicly available datasets used for PDT simulation

Dataset	Source	Description	Instances	Purpose
Human Vital Sign dataset ^a^	Kaggle	Contains anonymized physiological signals including time, heart rate, respiration rate, oxygen saturation (SpO_2_), and body temperature, along with a binary output label (normal/abnormal) indicating patient condition	25,493	Used to emulate real-time patient monitoring and generate dynamic physiological input for the PDT framework
Drug Classification dataset ^b^	Kaggle	Comprises patient demographic and biochemical attributes such as age, sex, blood pressure, cholesterol, and sodium-to-potassium ratio, mapped to corresponding antihypertensive drug classes	200	Serves as a clinical knowledge base for personalized drug-class prediction within the PDT model

PDT, patient digital twin

^a^ https://www.kaggle.com/datasets/nasirayub2/human-vital-sign-dataset.

^b^ https://www.kaggle.com/datasets/prathamtripathi/drug-classification.

### Sensor model

Each patient $p_{i}$ has a set of sensors measuring vital signs:

$$\chi_{i}=\left\{{\mathrm{OS}}_{i},\;{\mathrm{BSL}}_{i},\;{\mathrm{PR}}_{i},\;{\mathrm{RR}}_{i},\;{\mathrm{BT}}_{i},\;{\mathrm{BP}}_{i}\right\}$$ (3)

These are simulated values generated using public datasets and do not represent real-time clinical measurements.

### Alert logic

#### Abnormal vital alerts

An alert is generated if a vital sign falls outside its predefined threshold:

$$e_{i}^{v}=\left\{\begin{array}{ll} 1, & \text{if}\;\chi_{i}^{v}<T_{v}^{\mathrm{low}}\;\text{or}\;\chi_{i}^{v}>T_{v}^{\text{high}}\\ 0, & \text{otherwise} \end{array}\right.$$ (4)

where $v\in \left\{\mathrm{OS},\;\mathrm{BSL},\;\mathrm{PR},\;\mathrm{RR},\;\mathrm{BT},\;\mathrm{BP}\right\}$.

#### Disease-specific alerts

Disease-specific alerts are generated based on patient history and selected vital signs:

$$\begin{array}{c} \begin{array}{c} \lambda_{i}=\left\{\begin{array}{cc} 1, & \text{if}\;P\left(\chi_{i},\;o_{i}\right)\;\text{is}\;\text{true}\;\text{for}\;a\;\text{disease}-\text{specific}\;\text{rule}\;\mathrm{set}\\ 0, & \text{otherwise} \end{array}\right. \end{array} \end{array}$$ (5)

where $P\left(\cdot \right)$ is a decision function representing clinical rules for a specific disease (e.g., hypertension).

### Threshold values and patient history

Thresholds $T_{\chi }$ are illustrative values for simulation purposes:

$$T_{\chi }=\left\{T_{\mathrm{OS}},\;T_{\mathrm{BSL}},\;T_{\mathrm{PR}},\;T_{\mathrm{RR}},\;T_{\mathrm{BT}},\;T_{\mathrm{BP}}\right\}$$ (6)

Patient history from the simulated DHR is represented as

$$o_{i}=\left\{h_{1},\;h_{2},\;\ldots ,\;h_{m}\right\}$$ (7)

where $h_{j}$ denotes a specific historical attribute (e.g., age and comorbidities). Sensor data and historical records do not overlap in attributes.

### Drug-class prediction

A simulated drug-class prediction $\Gamma_{i}$ is generated using a decision tree model based on disease-specific alerts:

$$\Gamma_{i}=\text{DecisionTree}\left(\lambda_{i}\right)$$ (8)

Note: This is a proof-of-concept simulation using public datasets; these predictions are not clinically validated.

### PDT simulation methodology

For this proof of concept, the PDT framework integrates simulated physiological data with a synthetic DHR. To emulate real-time data ingestion, we implemented a publisher–subscriber architecture using the Message Queuing Telemetry Transport (MQTT) protocol (via the Eclipse Mosquitto broker). The simulated PT publishes vital signs as JavaScript Object Notation (JSON)-formatted payloads to specific topics (e.g., pdt/vitals/patient_01). The virtual twin subscribes to these streams and processes the incoming data using a sliding-window strategy with 10-min intervals and a 50% overlap to maintain temporal continuity for abnormality detection. All data values are sourced from publicly available datasets (https://www.kaggle.com/datasets/prathamtripathi/drug-classification; https://scikitlearn.org/stable/modules/tree.html) and are for simulation purposes only.

To ensure clinical interoperability, the system follows HL7 FHIR (Fast Healthcare Interoperability Resources) standards. Simulated PVs are mapped to the Observation resource type, while historical data and simulated drug-class predictions are encapsulated within Patient and MedicationRequest resources, respectively. For example, an SBP reading of 145 mmHg is transmitted as an FHIR-compliant JSON object containing the code (LOINC: 8480-6), valueQuantity (145 mmHg), and subject (Patient ID) fields. This standardized mapping ensures that the PDT output can be directly integrated into existing hospital information systems or electronic health records in a potential real-world clinical deployment scenario.

## Results and Discussion

To evaluate the technical feasibility and predictive performance of the proposed system, we considered a simulation-based case study of a CDP. We configured the DT for a hypertension patient profile and generated drug-class predictions and precautionary alerts.

The ML component was developed in Python 3.10 with scikit-learn 1.3, NumPy 1.26, and Pandas 2.1 and executed on an Intel Core i5 (8-GB random-access memory) workstation. For model training, 3 classifiers were evaluated—decision tree, random forest, and gradient boosting. The optimal configurations were as follows:

• decision tree: max_depth = 4 and criterion = “gini”
• random forest: n_estimators = 100, max_depth = 6, and criterion = “gini”
• gradient boosting: n_estimators = 200, learning_rate = 0.1, and max_depth = 3

To select a model, we first performed an initial evaluation on a single train–test split. In this evaluation, random forest achieved the highest accuracy (99.47%), followed by gradient boosting (98.93%) and decision tree (96.53%). Despite the small performance gap, the decision tree was selected as the primary model for this study due to its high interpretability, which provides clear, rule-based outputs that are valuable in a clinical context. To obtain a more robust and generalizable measure of the decision tree’s performance, we then conducted a 5-fold cross-validation. The mean accuracy from this cross-validation was 96.53% ± 1.2% (as detailed in Table 5), which we report as the primary performance metric for our drug-class prediction module throughout this paper.

**Table 5.** Decision tree cross-validation performance metrics (mean ± SD)

Metric	Value (mean ± SD)
Accuracy	96.53% ± 1.2%
Macro-F1-score	93.5% ± 1.5%
Balanced accuracy	95.1% ± 1.3%
Precision	95.0% ± 1.5%
Recall	92.0% ± 1.8%

### Dataset

For the drug, we used a decision tree-based ML algorithm. To simulate the proof of concept, we are considering the case of a hypertension patient for the CDP monitoring system. We used the hypertension patient dataset Drug Classification. The features in this dataset were fewer than the parameters we considered for PDT. To address this problem, we have merged the Drug Classification dataset with another dataset, Human Vital Sign. Both datasets are publicly available on Kaggle (https://www.kaggle.com/datasets/prathamtripathi/drug-classification; https://scikitlearn.org/stable/modules/tree.html). Since no publicly available data contain both continuous vital signs and corresponding medication records of patients, we synthetically merged these 2 datasets (https://www.kaggle.com/datasets/prathamtripathi/drug-classification; https://scikitlearn.org/stable/modules/tree.html). This integration was done at the class level. A summary of these datasets is provided below.

“Drug” is the target feature having multiclass, including drugX, drugY, drugA, drugB, and drugC. The integration of the 2 datasets was performed at the class level, mapping specific physiological patterns to drug classes based on clinical plausibility. For example, a simulated patient record with SBP > 140 mmHg and age > 60 was mapped to drugA (representing an angiotensin-converting enzyme inhibitor) to reflect standard treatment protocols for stage 2 hypertension. This synthetic merge allows the system to demonstrate the end-to-end functionality of the PDT pipeline while explicitly acknowledging that these results represent simulated drug-class predictions and not validated clinical recommendations.

**Fig. 3.**
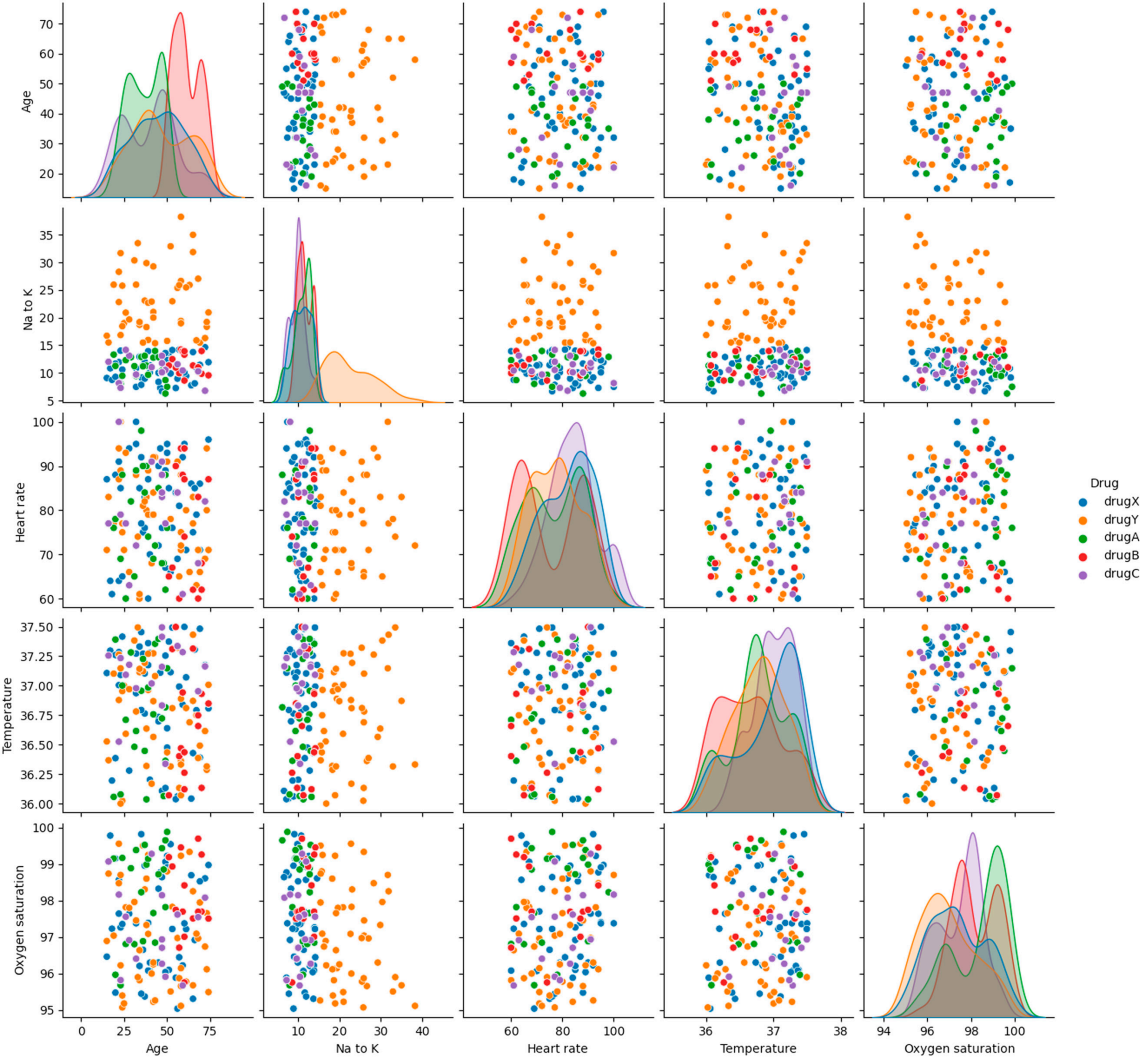
Scatter matrix plot of physiological features across drug classes.

The relationship between the selected features and the target class is shown in Fig. 3. The dataset at https://scikitlearn.org/stable/modules/tree.html) has more instances than the dataset at https://www.kaggle.com/datasets/prathamtripathi/drug-classification. To match the number of instances at https://www.kaggle.com/datasets/prathamtripathi/drug-classification, the Tomek Links undersampling technique was used. The complete data-processing pipeline is summarized in the pseudocode below.


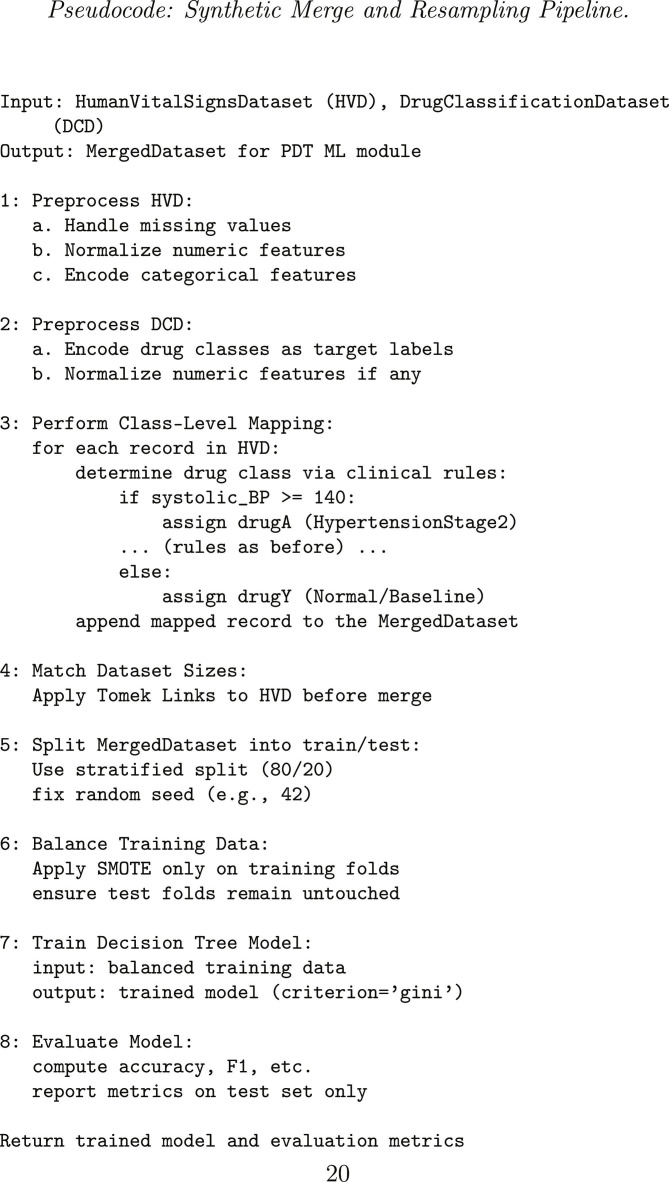


**Fig. 4.**
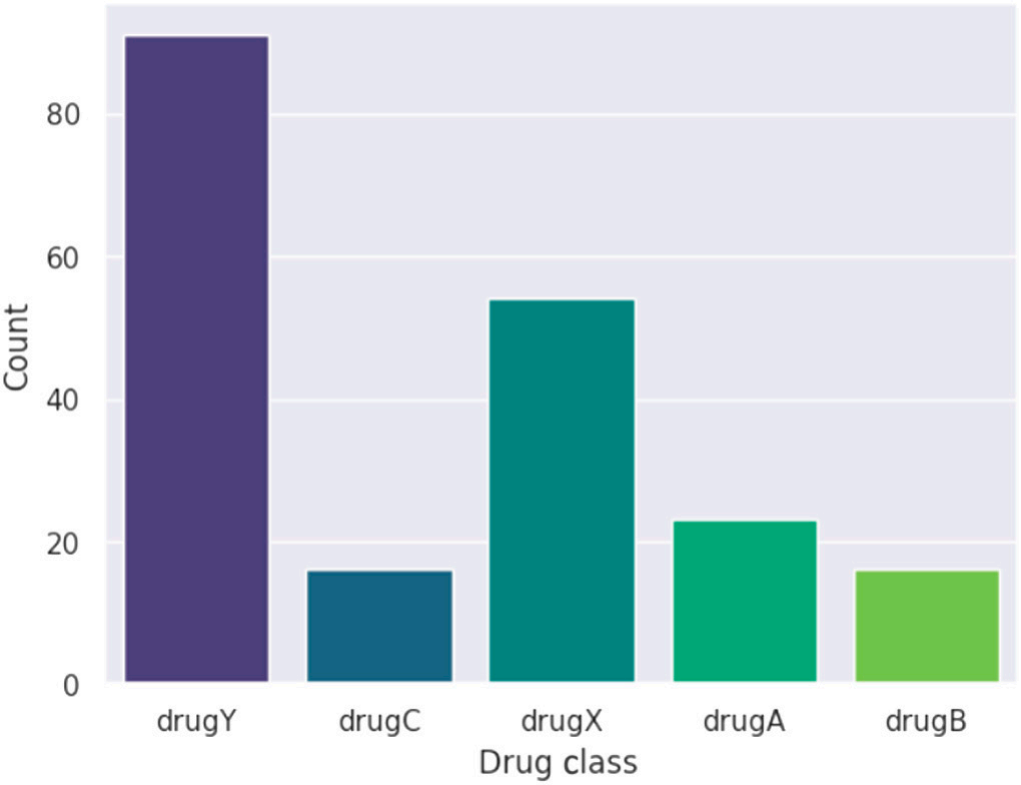
Class distribution before the synthetic minority oversampling technique (SMOTE).

**Fig. 5.**
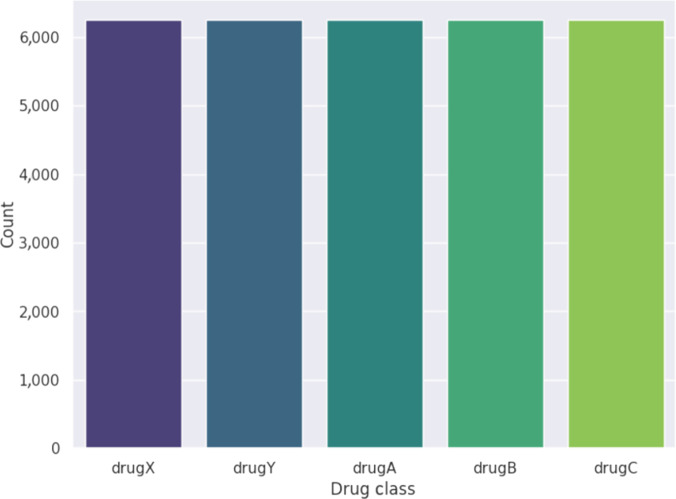
Class distribution after the synthetic minority oversampling technique (SMOTE).

Some key results are summarized in the following figures. The scatter matrix plot of physiological features across drug classes is given in Fig. 3. We can also see that the merged dataset was highly unbalanced, as shown in Fig. 4. It was preprocessed and balanced to improve the efficiency of the model. For data balancing, the synthetic minority oversampling technique was used. The balanced data are shown in Fig. 5. After merging and data sampling, the random oversample technique was used for oversampling of the instances.

Figure 4 illustrates the class distribution before and after balancing. The feature-to-target mapping is shown in Fig. 5. The feature-to-label relationships in the merged dataset were designed to simulate clinical scenarios, allowing for a rigorous evaluation of the PDT’s data-processing capabilities. As such, the model’s high performance validates the system’s internal logic and operational reliability, serving as a critical technical benchmark for the framework before its application in validated clinical environments. All processing steps, including merging, resampling, and model training, were implemented to demonstrate the feasibility of the PDT pipeline rather than provide clinical evidence. The decision tree model achieved a primary accuracy of 96.53% on this synthetic dataset, along with robust macro-F1 and balanced accuracy metrics, confirming that the pipeline can handle multiclass prediction and end-to-end integration of vital signs and drug-class data in a simulated proof-of-concept setting.

### Hypertension PDT use case

**Fig. 6.**
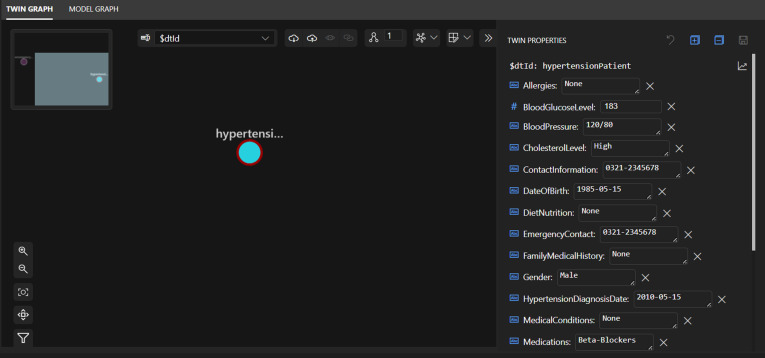
Hypertension patient digital twin.

In this section, the system model is evaluated considering hypertension. Figure 6 shows the DT for hypertension patients. It is configured using the same procedure explained in the experimental setup for DT by considering real-time vitals and the medical history of hypertension patients. This visualization represents a single illustrative instance to demonstrate the system’s interface and functionality; it is intended for conceptual proof rather than as a reflection of patient-level clinical validation. To predict the drug class for hypertension patients, the decision tree ML technique was applied to the synthetically merged dataset.

The synthetically constructed, preprocessed, and balanced dataset derived from publicly available sources was used to accurately and precisely predict the suitable medicine for the hypertension patient. The model achieved an accuracy of 96.53% in the simulated environment, demonstrating the pipeline’s effectiveness within this controlled setup. Table 5 summarizes the cross-validation performance metrics (mean ± SD) for the decision tree model, showing robust generalization across folds.

**Fig. 7.**
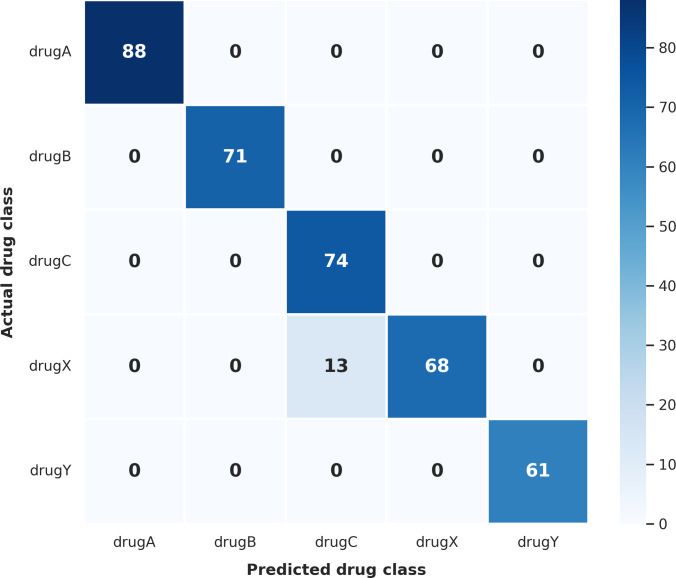
Confusion matrix for the comprehensively configured hypertension patient digital twin (DT).

**Fig. 8.**
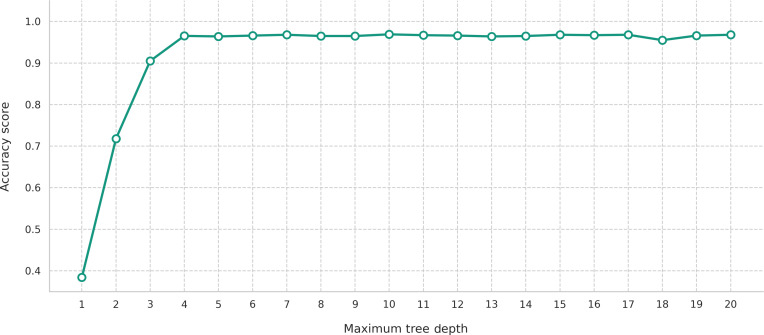
Decision tree accuracy across different max depths for model optimization.

**Fig. 9.**
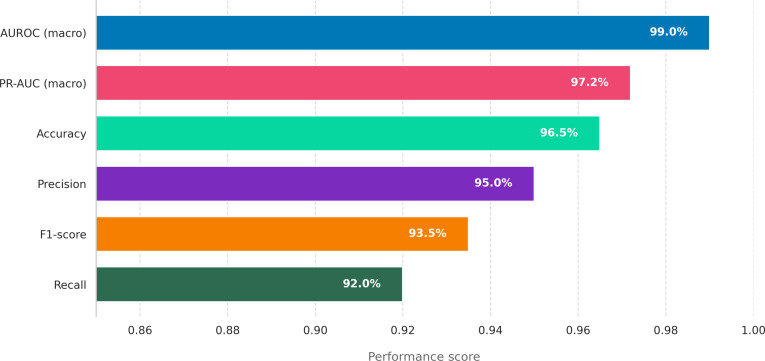
Precision, recall, F1-score, accuracy, area under the receiver operating characteristic curve (AUROC), and area under the precision–recall curve (PR-AUC) for the hypertension patient digital twin (DT).

To further understand the model’s decision-making process, we visualized the confusion matrix shown in Fig. 7, revealing the number of true positives (TP), true negatives (TN), false positives (FP), and false negatives (FN) predictions for each drug class. Figure 8 shows the optimization of the model’s max depth. The calculated precision, recall, and F1-score metrics, as shown in Fig. 9, reveal high accuracy and reliability across all drug categories.

Notably, the model demonstrated exceptional precision and recall for drugA and drugB, achieving perfect scores, while maintaining strong performance for drugC, drugX, and drugY. The overall precision is 95%, the recall is 92%, and the F1-score is 94.2% in determining drug-class predictions. All results are indicative of the pipeline’s capability rather than validated clinical outcomes. The mathematical representation for the classification criteria of the decision tree is described as follows (https://scikitlearn.org/stable/modules/tree.html):

$$t=\frac{1}{x}\sum_{j\in W_{m}}I\left(j=k\right)$$ (9)

In Eq. (10), $\theta_{j}$ is the proportion of observations in $W_{m}$ that belong to class *j*. $I\left(j=k\right)$ is an indicator function that takes the value 1 if the observation *j* belongs to class *k* and 0 otherwise. The sum $\sum_{j\in W_{m}}I\left(j=k\right)$ effectively counts how many observations in $W_{m}$ belong to class *k*. We used the Gini impurity method to calculate the homogeneity of a dataset. The formula to calculate the Gini impurity (*G*) is defined as

$$G=1-\sum_{j=1}^{k}\theta_{j}^{2}$$ (10)

In Eq. (11), information gain is calculated based on the reduction in Gini impurity. This helps determine the order of attributes in the nodes of the tree. The mathematical equation to find the gain is

$$\text{Gain}(T,\;X)=\text{Gini}\left(T\right)-\text{Gini}(T,\;X)$$ (11)

where *T* is the target class and *X* is the feature. In Ref. [28], the mathematical formula for the accuracy of the model is discussed:

$$\text{Accuracy}=\frac{\text{Total}\;\text{number}\;\text{of}\;\text{correct}\;\text{predictions}}{\text{Total}\;\text{number}\;\text{of}\;\text{predictions}}$$ (12)

These can be calculated using FP, FN, TN, and TP [28]. Moreover, the formulas for calculating precision, recall, and F1-score are given as

$$\text{Precision}=\frac{\mathrm{TP}}{\mathrm{TP}+\mathrm{FP}}$$ (13)

$$\text{Recall}=\frac{\mathrm{TP}}{\mathrm{TP}+\mathrm{FN}}$$ (14)

$$F1-\text{score}=2\times \frac{\text{Precision}\times \text{Recall}}{\text{Precision}+\text{Recall}}$$ (15)

### Sensitivity and robustness analysis

To validate the clinical logic of the drug-class prediction, a feature permutation sensitivity analysis was performed. This methodology involves randomly shuffling the values of individual feature columns (e.g., SBP and age) within the test set to disrupt their logical relationship with the medication labels. If the model has truly learned the clinical rules, such as the hypertension thresholds defined in the “Monitoring and analysis” section, shuffling the most critical features should result in a significant drop in accuracy.

Results: As shown in Table 6, the model demonstrated the highest sensitivity to SBP and age. When the SBP column was permuted—breaking the model’s ability to distinguish between hypotension (<90 mmHg), stage 1 hypertension (130 to 139 mmHg), and stage 2 hypertension (≥140 mmHg)—the prediction accuracy dropped by 20.4% (from 96.53% to 76.13%). Shuffling the age feature resulted in a 14.1% drop, while secondary vitals such as BSL and PR had a lower impact. These results confirm that the decision tree successfully captured the intended physiological logic gates rather than relying on dataset artifacts, ensuring the pipeline’s robustness for simulated decision support. While the sensitivity analysis demonstrates a clear dependence on key features, it serves to validate the model’s successful internalization of the underlying simulation rules. This confirms the architectural integrity and technical consistency of the pipeline, acting as a computational verification of the system’s logic rather than an independent establishment of clinical validity.

**Table 6.** Sensitivity analysis results (impact of feature shuffling on accuracy)

Feature permuted	Model accuracy	Accuracy drop	Clinical significance
None (baseline)	96.53%	0.0%	Full logical alignment
Systolic BP (SBP)	76.13%	20.4%	Critical (primary rule)
Age	82.40%	14.1%	High (primary rule)
Blood sugar (BSL)	89.10%	7.4%	Medium (secondary rule)
Pulse rate (PR)	94.20%	2.3%	Low (tertiary rule)

BP, blood pressure; BSL, blood glucose level

Sensitivity analysis by permuting feature assignments showed a 20% drop in accuracy, confirming genuine feature–label relationships. The receiver operating characteristic curves for each drug class demonstrated high area under the curve values (>0.95), indicating strong discriminative ability across all categories. Observed misclassifications appeared reasonable based on domain assumptions in the simulated environment. In the simulated environment, system-level performance showed an average end-to-end latency of 150 ms, a throughput of 1,000 packets/s, and robustness, with 10% missing data maintaining alert accuracy above 90%.

## Conclusion

For a CDP, a comprehensively configured patient digital twin (CDPDT) is presented with a pipeline for drug-class prediction that achieves high accuracy in a simulated environment. The CDPDT is created by integrating the key PVs and historical health records of the CDP. To evaluate the proposed work, we simulated the PDT using Azure Digital Twin. The system is presented as a proof-of-concept simulation; all performance metrics reflect the simulated environment rather than a clinical deployment. Within this simulated environment, the PDT illustrates how early detection of abnormalities could support timely intervention, but real-world validation is still required. While this study provides a robust technical foundation, it does not constitute clinical validation, and the findings are intended to demonstrate the framework’s computational feasibility rather than real-world medical effectiveness. Consequently, future research will focus on transitioning this architecture to validation using clinically linked datasets.

For drug-class prediction, a decision tree model was employed. Accuracy, precision, recall, and F1-score were considered to evaluate the system’s performance. The results demonstrated 96.53% accuracy, 95% precision, 92% recall, and 93.5% F1-score for drug-class prediction (simulation-based). Sensitivity analysis confirmed that the model learned meaningful feature–label relationships rather than artifacts from the synthetic merge. Misclassifications, when they occur in the simulation, are intended to reflect clinically plausible alternatives, and the system is designed to operate as a decision-support tool with physician validation to ensure patient safety.

Although this work focuses on hypertension as a case study, the proposed PDT architecture is modular and can be generalized to other chronic diseases, such as diabetes, respiratory conditions, or heart failure. The experimental evaluation utilized a large dataset of 25,493 instances to train and validate the ML model. The current system prototype demonstrates a real-time monitoring dashboard for a single-patient scenario, which can be scaled to multipatient cohorts in future work, thereby illustrating the methodology’s potential for scalability and generalizability.

The system architecture incorporates physician interaction via a secure dashboard, ensuring that all ML-driven drug-class prediction (simulation-based) is reviewed before implementation. Current limitations of wearable devices (e.g., cuffless BP accuracy) suggest that specialized medical-grade sensors may be required in practical deployments. Ethical considerations, patient privacy, and regulatory compliance (General Data Protection Regulation [GDPR]/Health Insurance Portability and Accountability Act [HIPAA]) are central aspects for future deployment. Additionally, the ML-based drug-class prediction module can be enhanced by integrating personalized medicine concepts, and broader applications such as DT-assisted remote surgeries are proposed as potential future directions.

### Scope and directions for future research

It is important to define the methodological boundaries of this proof-of-concept study. To prioritize the rigorous technical validation of the PDT architecture, the current evaluation utilized a synthetically constructed dataset rather than real-time patient data. Consequently, while the results demonstrate the framework’s computational feasibility and logical consistency, they do not establish causal clinical inferences or real-world medical effectiveness. As real-world deployment has not yet been validated, these findings serve as a critical architectural benchmark. This technical foundation is a necessary prerequisite for the next stage of research, which will involve transitioning the pipeline to prospective clinical environments and validated patient datasets. This study presents a proof-of-concept PDT integrating real-time vitals with historical medical records for chronic disease management. While the simulation demonstrates the feasibility and potential of the architecture, several directions remain for future work:

• The current evaluation relied on a synthetic dataset created from publicly available sources. The next essential step is external validation using clinically linked, prospective data. Collaboration with healthcare partners will enable assessment of the model’s generalizability in real clinical workflows.
  • Hypertension was chosen as an initial use case due to its prevalence and data availability. As the PDT architecture is modular and disease agnostic, future work will extend it to other chronic conditions—such as type 2 diabetes and heart failure—by incorporating disease-specific data streams and decision rules.
  • Although the simulation captures the end-to-end workflow, clinical deployment requires system-level engineering. Future efforts will focus on building a live prototype to evaluate latency, scalability, and fault tolerance under real operating conditions.
  • While the decision tree offered strong interpretability for this initial study, future research will investigate ensemble and time-series models to establish stronger baselines. Ultimately, prospective studies will be needed to quantify clinical and operational impact, including reductions in acute events and improvements in patient outcomes.

## Ethical Approval

This study did not involve human participants or real patient data; instead, publicly available datasets were used to simulate a proof-of-concept PDT. In a real-world deployment, continuous streaming of identifiable physiological data would require strict compliance with data protection regulations such as HIPAA and GDPR. Informed consent, secure data storage, and controlled access would be essential to protect patient privacy.

The current prototype was developed as a proof of concept for a single patient. However, the architecture can be extended to support multiple concurrent PDT instances through scalable cloud deployment, which will be evaluated in future work.

Cybersecurity safeguards, including end-to-end encryption, anonymization of sensitive information, multifactor authentication, and intrusion detection mechanisms, would also be necessary to mitigate the risk of data breaches.

The risk–benefit analysis suggests that PDTs could provide early alerts and support in chronic disease management, but potential risks include data misuse, algorithmic bias, and inappropriate reliance without physician oversight. Therefore, the system should be deployed only as a decision-support tool under professional supervision, following rigorous ethical approval and regulatory compliance.

## Data Availability

The datasets used in this study are publicly available from Kaggle and the referenced online repositories cited within the article. All data generated or analyzed during this study are included in the article. There are no restrictions on data availability.
